# Loop Myopexy Surgery for Strabismus Associated with High Myopia

**DOI:** 10.1155/2016/8657036

**Published:** 2016-04-27

**Authors:** Yun Su, Qin Shen, Xianqun Fan

**Affiliations:** Department of Ophthalmology, Shanghai Ninth People's Hospital, Shanghai Jiao Tong University, School of Medicine, Shanghai 200011, China

## Abstract

Strabismus associated with high myopia is a rare abnormality of ocular motility, leading to the impairment of abduction and supraduction. Loop myopexy of the superior rectus (SR) and lateral rectus (LR) muscles is now the most preferred surgery for restoring the dislocated eye globe back into the muscle cone. Various procedural modifications have been made based on this concept, and satisfactory outcomes have been reached in most cases. In this paper, we review various surgical modifications published in the literature that are based on the loop myopexy surgery in patients with high myopic strabismus and summarize the applicable scope of different surgical procedures for patients with different degrees of strabismus. Three major surgical procedures are identified and different modifications have been applied based on their concept. Most of these modifications have been proven to be safe and effective and result in good ocular alignments. The selection of such modifications is of great importance in different patients. Careful evaluation before surgery should be made not only to make the correct diagnosis but also to choose an appropriate surgical procedure and offer individualized modifications in the surgery.

## 1. Introduction

Strabismus associated with high myopia is a rare abnormality of ocular motility characterized by the presence of esotropia and hypotropia, with restricted abduction and supraduction [1]. In later stages, the affected eye progresses into an extreme esotropic and hypotropic position and the ocular motility is seriously limited, a condition called “myopic strabismus fixus” [2, 3].

With the evolving understanding of the pathogenesis, consensus has now been reached that strabismus with high myopia is usually the consequence of the supertemporal protrusion of the elongated eye globe through the muscle cone, leading to an inferior displacement of the lateral rectus muscle and nasal displacement of the superior rectus muscle [3, 4]. In addition, Rutar and Demer [5] found that degeneration in orbital connective tissues, usually the band bridging the lateral and superior rectus muscles, also enables a supertemporal shift of the globe through the weak ligament.

Based on the updated pathogenetic mechanism, the traditional recession-resection surgery, which aimed at altering the forces of the muscles, is no longer popular due to its limited effects, especially in some severe cases [1, 3]. Many surgical procedures aim at correcting the muscle paths of both the superior and lateral rectus muscles. Loop myopexy of the superior rectus (SR) and lateral rectus (LR) muscles is now the most preferred surgery for restoring the dislocated globe back into the muscle cone [6]. Various modifications have been made based on this surgical approach, and satisfactory outcomes have been reached in most cases [7–24].

In this paper, we review the various surgical management techniques published in the literature that are based on the procedure of loop myopexy for treating patients with strabismus associated with high myopia. We also summarize the applicable scope of surgical procedures for patients with different degrees of strabismus.

## 2. Advantages of Loop Myopexy Surgery

### 2.1. Traditional Surgical Management Techniques

Recession-resection surgeries have been traditionally applied to alter the forces of the muscles. Hayashi et al. [1] and Louis et al. [25] performed recession of the medial rectus (MR) muscle and resection of the LR in patients in the early stage of strabismus and obtained good results. However, the correction was not enough for more severe cases. Krzizok et al. [3] claimed that the recession-resection procedure might aggravate the deviation because the dislocation of the LR anatomically would reduce its abducting force and create depressing forces. They advocated that the most important objective of surgical management is to normalize the pathological path of the LR instead of reinforcing its forces. They proposed a new surgical technique that dislocated the LR to the physiological meridian at the equator with a nonabsorbable suture or silicon loop, combined with a large recession of the MR and LR. However, their procedure was technically difficult and had a high risk of globe perforation due to the thin sclera in high myopia [26].

### 2.2. Loop Myopexy Surgery

In the past, most attention has been paid to the management of the horizontal muscles because some researchers claimed that it was the dislocation of only the LR by the elongated eyeball that caused the corresponding strabismus [1, 3]. However, this concept was updated by Yokoyama's study [6]. After an evaluation of the anatomic relationships between the muscle cone and globe on magnetic resonance imaging (MRI), they found that the posterior portion of the elongated globe was dislocated from the muscle cone supertemporally, leading to an inferior shift of the LR and nasal shift of the SR. Later, these findings were further confirmed by Aoki et al. [4] by investigating the extraocular muscle path shift and prolapse of the posterior eyeball from the muscle cone. They also agreed that it was the posterior portion of the globe that stretched and shifted both of the muscles.

Based on this hypothesis, Yokoyama et al. proposed a loop myopexy surgery of the muscle bellies of the SR and LR to restore the dislocated globe back into the muscle cone [6]. This surgery united these two muscles and reestablished the physiological muscle plane, thus preventing the globe from prolapsing through the supertemporal quadrant [6, 17, 20]. Various modifications have been suggested on the basis of uniting these two muscles. Most patients had satisfactory results with good ocular alignment.

Yamada's procedure, a hemitransposition of the SR and LR, cannot be strictly included as a loop myopexy surgery because it merely secures the muscle bellies to the sclera without actual union of these two muscles [17]. However, its concept was similar to that of a loop myopexy surgery in that it tried to change the position of the SR and LR and reconstruct the normal anatomic relation of the muscles. Moreover, the application of Yamada's procedure and its modifications showed good postoperative results in patients with strabismus fixus [17–19]. Therefore, in this review, we also included it as a special type of loop myopexy surgery.

## 3. Procedures of Loop Myopexy Surgery

All studies regarding loop myopexy in high myopic strabismus in the literature were reviewed and the main surgical procedures were determined. Eighteen studies of strabismus associated with high myopia treated with the procedure of loop myopexy were eligible for our review. The clinical characteristics, surgical management, and outcomes of eighty-nine patients, with ages ranging from 5 to 78 years, are described in Table 1.

Three main surgical management techniques were found in the literature, namely, Yokoyama's procedure, Yamada's procedure, and the partial Jensen's procedure. Other studies found in the literature were performed based on these three procedures, with modifications in various details of the surgery, such as being with or without medial rectus (MR) muscle recession, scleral fixation, or the use of materials for muscle union. All three surgical procedures were based on the concept of pathogenesis described by Yokoyama et al. [6].

### 3.1. Yokoyama's Procedure

Yokoyama's procedure, first performed in 2000, has drawn wide attention because of its satisfactory postoperative ocular alignment and the concept that there is no abnormality of the muscle forces but abnormality of the muscle paths [6, 10, 26]. In his study, six patients with high myopic strabismus were treated with a full loop myopexy of the SR and LR muscle belly 15 mm behind the insertions using a polyester suture. Postoperatively, they achieved great improvement in ocular motility and reduced herniation of the globe, as confirmed by MRI [10]. Later, in nine studies, a total of 48 patients were treated using this full loop myopexy technique with modifications of various degrees [7–16].

### 3.2. Yamada's Procedure

In 2002, Yamada et al. [17] performed a hemitransposition of the SR and LR, combined with a large recession of the MR in a patient with bilateral convergent strabismus fixus. They divided the SR and LR in half 15 mm from the insertion, secured the temporal half of the SR and the superior half of the LR to the sclera between the SR and LR at 7 mm posterior from the limbus, and performed a recession of the MR by 8 mm. Based on this procedure, Sturm et al. [18] tied the translocated muscle halves together and secured them to the supertemporal sclera at 15 mm from the new insertion. Godeiro et al. [19] applied the hemitransposition technique, together with MR recession or Botox injection.

### 3.3. Partial Jensen's Procedure

The partial Jensen's procedure was first performed by Larsen and Gole in 2004 [20]. They split the SR and LR muscles in half, from the insertion to past the equator, and apposed only the adjacent halves of the LR and SR muscles. After surgery, patients achieved significant improvement in ocular motility and good cosmetic results. A total of 15 patients with high myopic strabismus in four other studies received similar surgical treatment [21–24].

## 4. Modifications of Loop Myopexy Surgery

In other studies, various modifications have been performed based on the three surgical procedures mentioned above (i.e., Yokoyama's, Yamada's, and partial Jensen's). To eliminate the risk of scleral perforation, some authors preferred not to suture the muscle bellies onto the globe [7–15, 20–24]. In some studies, a hang-back technique that did not touch the sclera was also preferred in the MR recession [18, 24]. To minimize the possibility of anterior segment ischemia, some authors advocated a union of parts of the muscle belly because the unsecured parts of the SR and LR muscles would contribute to the circulation of the anterior segment [20–22]. Moreover, given the potential complications of muscle cheese-wiring and the disadvantage of the irreversibility of suture loop myopexy, different materials, mainly silicone bands, have been applied in the surgery [7, 12, 16]. Shenoy et al. [16] performed a novel modification of loop myopexy with a silicone band in 15 patients with high myopic strabismus. They believed that there was an increased risk of migration of the silicone band, especially in eyes with great axial length, so they advocated the scleral fixation of the band. This technique was proven to be effective and can improve alignment significantly. However, two patients in that study presented with complications of foreign body sensation, which required removal of the silicone band.

## 5. Selection of Surgical Procedure

### 5.1. Selection of a Surgical Procedure Based on Degrees of Strabismus

**Figure 1:**
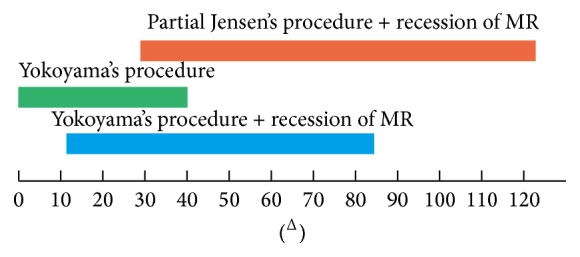
Applicable scopes of different surgical procedures for the treatment of high myopic strabismus. Yokoyama's procedure alone can correct up to 40^Δ^ of esotropia. A combination of Yokoyama's procedure and recession of the MR muscle is effective in patients with 12 to 85^Δ^ of esotropia. The partial Jensen's procedure combined with recession of the MR muscle is able to correct esotropia over 30^Δ^.

The selection of a surgical procedure is based mainly on the surgeon's preference and proficiency in a certain technique. All three procedures discussed above have been proven to be effective in the treatment of high myopic strabismus. However, there exist some differences in the applicable scope of each procedure, which is a consideration that might be beneficial when planning a surgery. Shenoy et al. found that full loop myopexy of the SR and LR alone can correct up to 40^Δ^ of esotropia [16]. After a thorough review of the application of Yokoyama-based procedures (i.e., full loop myopexy), we found it to be more effective in patients with esotropia of 12 to 85^Δ^ when combined with recession of the MR muscle [10, 15]. Furthermore, the partial Jensen's procedure combined with MR recession has a wider range of application; patients with large esotropia, usually over 90^Δ^, also have satisfactory outcomes after surgery [21, 24]. Because the data in studies related to Yamada's procedure and the simple partial Jensen's procedure were limited, we are not able to summarize the applicable scope of these two procedures. Based on the existing findings, we created a diagram for the applicable scopes of different surgical procedures for the treatment of high myopic strabismus (Figure 1). However, these scopes were derived mainly from the summary of our literature review. Further studies of more patients are warranted to verify the accuracy of these applicable scopes.

### 5.2. Application of Surgical Procedures to Fixed Eye Position

Myopic strabismus fixus is an extreme condition in which the affected eye is fixed inferomedially with restricted ocular motility. The elongated globe is dislocated from the muscle cone, and the posterior portion of the globe is blocked by the SR and LR, thus leading to restriction in abduction and sursumduction. The main purpose of surgery is to eliminate the mechanical limitation in ocular motility.

Hayashi et al. classified patients with high myopic strabismus into four groups, and the surgical management of patients who presented with fixed eye in the extreme position was considered a great challenge [1]. Basmak et al. performed Yokoyama's procedure together with recession of the MR in a 55-year-old female patient with both eyes fixed in the extreme esotropic and hypotropic position [9]. Postoperatively, good outcomes were achieved despite mild restriction in all directions. Yamada's hemitransposition procedure combined with the MR recession was performed in a 69-year-old patient with fixed eye positions and limited ocular motility in both eyes [17]. One year after surgery, the patient's motility greatly improved, although there was mild impairment of abduction and elevation. Ho et al. treated two cases (one bilateral and one unilateral) of severe high myopic strabismus fixus by the partial Jensen's procedure combined with MR recession [24]. Good ocular alignments were achieved after surgery with less than 10^Δ^ esotropia in individual cases. Thus, all three surgical procedures mentioned above (i.e., Yokoyama's, Yamada's, and partial Jensen's) have been proven to be effective in the treatment of myopic strabismus fixus.

### 5.3. Role of the Additional MR Recession

The MR recession is not necessary in all patients. If forced duction test confirms tight muscle or if restricted abduction exists for several years, contracture of the MR muscle is likely to have occurred [27]. In these cases, the MR recession is recommended together with the union of the LR and SR [10, 14]. In the study of Akar et al. [14], they performed the MR recession at the same time as muscle union in 24 eyes of 13 patients whose preoperative forced duction tests were positive, and all of them had significant improvement in abduction and supraduction. However, if there is no evidence of MR contracture before surgery, the MR recession can be applied in a second surgery when the union of LR and SR is not sufficient to correct the abnormal muscle paths. Yamaguchi et al. [10] applied the same surgical strategy for the MR recession, and four patients in their study presented without evidence of MR contracture and were successfully cured by only uniting the LR and SR muscles.

### 5.4. Surgery of Bilateral High Myopic Strabismus

In patients with strabismus associated with high myopia in both eyes, we recommend that bilateral strabismus surgeries be performed simultaneously to correct the muscle paths, not only to eliminate the disparity between the two eyes but also to avoid the potential risk of having the patient undergo a second general anesthesia, especially in the elderly. In those severe cases, large MR recession is usually needed because some residual esotropia will remain after union of the SR and LR bellies. The MR recession will be helpful to further correct the eye position. Morad et al. held a similar opinion that bilateral myopexy of the SR and LR is the preferred method, after comparing the surgical results of patients with high myopic strabismus unilaterally and bilaterally [28]. If a unilateral myopexy surgery is selected, a procedure that also combines ipsilateral MR recession will contribute to satisfactory outcomes and decrease residual esotropia [29].

### 5.5. Process of Selecting a Surgical Procedure

**Figure 2:**
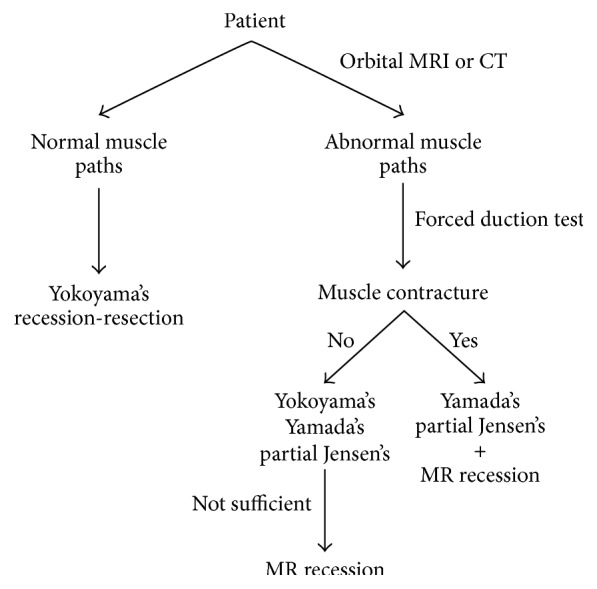
Flow diagram of the selecting process of surgical procedure. MRI or CT scan of the orbit before surgery can give a clear demonstration of the rectus muscle paths. If there is an absence of alteration in muscle paths, both Yokoyama's procedure and traditional recession-resection surgery have been recommended in some studies. In patients with abnormality in muscle paths, if contracture of the MR muscle is found, the MR recession is recommended together with the union of the LR and SR. If there is no evidence of MR contracture, the MR recession can be staged in a second surgery when the union of LR and SR is not sufficient to correct the abnormal muscle paths.

A thorough eye examination and imaging of the patient before surgery will be helpful for the surgical strategy. Patients are suggested to have an MRI or CT scan of the orbit, which can give a clear demonstration of the rectus muscle paths, the relationship between the globe and muscle cone, and also the shape of the globe to exclude thyroid eye disease [26], severe myopic staphylomata [29], or sagging eye syndrome [30, 31]. If there is an absence of alteration in muscle paths, both Yokoyama's procedure and traditional recession-resection surgery have been recommended in some studies [11, 18]. However, it is still a controversy in the selection of surgical procedures in these cases. When the imaging test has confirmed an abnormality in muscle paths, forced duction test can contribute to distinguishing contracture of the MR muscle. If contracture of the MR muscle is found, the MR recession is recommended together with the union of the LR and SR [10, 14]. When performing these two procedures together, there exists the risk of anterior segment ischemia when involving three muscles, so uniting half of the LR and SR muscles (Yamada's or Partial Jensen's procedure) would be preferred in combination with the MR recession [21, 23, 24]. On the other hand, if there is no evidence of MR contracture before surgery, the MR recession can be staged in a second surgery when the union of LR and SR is not sufficient to correct the abnormal muscle paths. Yokoyama's procedure has been proven to be effective to correct up to 40^Δ^ of esotropia [16]. Yamada's and Partial Jensen's procedures have been performed successfully in patients with around 40^Δ^ to 60^Δ^ of esotropia [19, 20, 22]. A flow diagram illustrates the selecting process of a certain surgical procedure (Figure 2).

## 6. Summary

Loop myopexy is a safe and effective surgical treatment in patients with high myopic strabismus. Different modifications of this surgical procedure can be applied based on its concept, most of which have been proven to have good surgical outcomes. The selection of such modifications is of great importance in different patients. Careful evaluation before surgery should be made not only to make the correct diagnosis but also to choose an appropriate surgical procedure and offer individualized modifications in the surgery.

## Figures and Tables

**Table 1:** Summary on literatures about surgical treatment of high myopic strabismus.

Procedures	Authors	Number of cases	Preoperative strabismus	Surgical management	Patients' outcome	Follow-up (months)
Yokoyama's procedure	Wong et al., 2005 [7]	2	Case 1: 70^Δ^ esotropia and 25^Δ^ hypotropia with limited ocular movement Case 2: fixed eye position in adduction and depression	Union of the SR and LR 12 mm behind the limbus Case 1: with a 5-0 Mersilk suture Case 2: with a 240 band and silicon sleeve	Case 1: 10^Δ^ esotropia with mild limitation in abduction Case 2: 14^Δ^ exotropia, limitation in abduction (−1) in the right eye, adduction (−2) in the left eye, and elevation (−1) in both eyes	Case 1: 15 Case 2: 14
	Rowe and Noonan, 2006 [8]	1	70^Δ^ esotropia and 25^Δ^ hypotropia	Union of the temporal half of SR and the upper half of LR, placed posterior to the equator by 5-0 polybutilate-coated polyester suture; recession of the MR for 6 mm	20^Δ^ hypotropia, mild limitation of elevation and abduction	12
	Basmak et al., 2008 [9]	1	Fixed eye position and restricted ocular motility	Union of the SR and LR 15 mm behind their insertions with a nonabsorbable polyester suture; recession of the MR 12 mm from the limbus	Restoration of the dislocated eyeball and improvement of ocular motility, but mild restriction in all gaze directions	24
	Yamaguchi et al., 2010 [10]	21	Angles of deviation 58.8 ± 36.0°	Union of the SR and LR 15 mm behind the insertions with a polyester suture; recession of the MR for 5 to 8 mm	Angles of deviation of 0.7 ± 9.0° degrees, great improvement in abduction and sursumduction	48.8
	Durnian et al., 2010 [11]	5	13^Δ^ esotropia and 21.8^Δ^ hypotropia	Union of half of the SR and LR 14 mm behind their insertions with 5-0 nonabsorbable suture	Hypotropia deviation of 0^Δ^ and vertical deviation of 4.4^Δ^	6
	Shih et al., 2012 [12]	1	Severe limitation of ductions in all directions	Union of the SR and LR 5 to 7 mm posterior to their insertions with a polytetrafluoroethylene (Gore-Tex) Sling and 5-0 polyester suture; recession of the MR for 8 mm and LR for 6 mm	12^Δ^ esotropia with limitation in supraduction (−3)	7
	Akbari et al., 2013 [13]	1	Fixed eye position in adduction and infraduction	Union of the SR and LR with a polyester suture; recession of the MR and resection of LR	Binocularly aligned	12
	Akar et al., 2014 [14]	20	58.6 ± 2.5^Δ^ esotropia and 12.5 ± 1.3^Δ^ hypotropia	Union of the lateral one-quarter of the SR and the superior one-quarter of the LR 14 to 15 mm posterior to the insertions with a double-armed 5-0 polybutilate-coated polyester suture; recession of the MR for 8 to 10 mm	6.8 ± 1.4^Δ^ esotropia, 3.3 ± 1.1^Δ^ hypotropia, and significant improvement in abduction and supraduction	48
	Acar and Altintas, 2015 [15]	2	Case 1: 65^Δ^ esotropia Case 2: 85^Δ^ esotropia	Union of the SR and LR with a 5-0 nonabsorbable polyester suture; recession of the MR for 5.75 mm	Case 1: 16^Δ^ at near and distance Case 2: 12^Δ^ at near and 14^Δ^ at distance, mild limitation in abduction (−1)	Case 1: 36 Case 2: 25
	Shenoy et al., 2015 [16]	15	79.3 ± 32.3^Δ^ esotropia and 8.9 ± 10.1^Δ^ hypotropia	Union of the SR and LR 14 to 16 mm from the limbus through a 3 to 4 length scleral tunnel with 240 silicone band and 5-0 nonabsorbable polyester suture; recession of the MR for 5 to 7.5 mm	16.9 ± 17.4^Δ^ esotropia, 0.6 ± 1.3^Δ^ hypotropia, and success rate (deviation ≤ 20^Δ^) 73%	7.9 ± 8.5

Yamada's procedure	Yamada et al., 2002 [17]	1	Fixed eye position in extreme adduction and restricted ocular motility	Hemitransposition of the SR and LR with scleral fixation 7 mm from the limbus; recession of the MR for 8 mm	10^Δ^ esotropia with mild limitation in supraduction and abduction	12
	Sturm et al., 2008 [18]	1	Fixed eye position in extreme adduction and depression	Hemitransposition of the SR and LR with a new insertion at 7 mm posterior from the limbus and myopexy of translocated muscles with scleral fixation at 15 mm from the new insertion; recession of MR for 10 mm	20° esotropia with slightly limited ocular motility in abduction, elevation, and adduction	12
	Godeiro et al., 2009 [19]	2	Case 1: 50^Δ^ esotropia and 12^Δ^ hypotropia Case 2: 60^Δ^ esotropia and 10^Δ^ hypotropia	4 mm resection and hemitransposition of the SR and LR with scleral fixation at 7 mm from the limbus by 6-0 mersilene Case 1: 5 IU botulinum toxin injection into the MR; then recession of the MR for 6 mm Case 2: recession of the MR for 6 mm	Case 1: satisfactory alignment with mild limitation in abduction and elevation at 8 months Case 2: excellent ocular alignment with a marked improvement in abduction and elevation	Case 1: 8 Case 2: 6

Partial Jensen's procedure	Larsen and Gole, 2004 [20]	1	50^Δ^ esotropia and 30^Δ^ hypotropia	Union of the lateral half of the SR and the superior half of the LR 14 mm from the limbus with 5-0 Dacron suture	Significant improvement in abduction and supraduction	9
	Ahadzadeghan et al., 2009 [21]	6	≫90^Δ^ esotropia and 25 to 30^Δ^ hypotropia	Union of the lateral half of the SR and the superior half of the LR 16 mm from the limbus with 5-0 Dacron suture; recession of the MR for 6 to 10 mm	5 to 20^Δ^ esotropia and mild limitation in abduction and elevation	2
	Rajavi et al., 2009 [22]	2	Case 1: 40^Δ^ esotropia and 5^Δ^ hypotropia Case 2: 50^Δ^ esotropia and 2^Δ^ hypotropia	Union of the lateral half of the SR and the superior half of the LR posterior to the equator with a 5-0 nonabsorbable polybutilate-coated polyester suture	Case 1: 10 to 12^Δ^ esotropia and 3^Δ^ hypotropia, slightly restricted abduction (−1) Case 2: 25^Δ^ for far and 20^Δ^ for near esotropia and 2^Δ^ hypotropia, mild restriction (−1) in abduction	Case 1: 3 Case 2: 2
	Kang et al., 2011 [23]	5	82.86 ± 37.62^Δ^ esotropia and 20 ± 7.91^Δ^ hypotropia	Union of the lateral half of the SR and the superior half of the LR 12 to 14 mm from the insertions with a 5-0 nonabsorbable polyester suture; recession of the MR for 6 to 10 mm	Significant improvement in dislocation of the globe, ocular motility, and horizontal and vertical deviations	5
	Ho et al., 2012 [24]	2	>90^Δ^ esotropia and marked limitation on abduction (−3)	Union of the lateral half of the SR and the superior half of the LR 14 mm from the limbus with a 5-0 Dacron nonabsorbable suture; recession of the MR for 8 to 8.5 mm	Case 1: great improvement in ocular motility and alignment with 10^Δ^ esotropia Case 2: improved motility with 4^Δ^ esotropia	Case 1: not mentioned Case 2: 10
